# Death from Twenty Grains of Chloral

**Published:** 1877-03

**Authors:** E. Fletcher Ingals

**Affiliations:** Chicago


### DEATH FROM TWENTY GRAINS OF CHLORAL.

Chicago, February 12, 1877.

To the Editor of the Chicago Medical Journal and Examiner: Dr. A. Ashbaugh has given me the following notes of a case of poisoning by chloral which he witnessed in the office of a friend, in Monville, Mo., about three years ago. The case has never been reported:

A German woman, about thirty-three years of age, apparently healthy, came into the office to have some teeth extracted. She desired some medicine to prevent the pain, and the doctor gave her ten grains of hydrate of chloral, which dose he repeated in one hour. Soon after the second dose the patient manifested alarming symptoms of poisoning, and although all was done that could be to resuscitate her, she died in about fifteen minutes. No *post mortem* examination was made. The patient had taken only two doses of the chloral, of ten grains each — the second having been given one hour after the first.

E. Fletcher Ingals, M.D.

### AN ELEGANT DISINFECTANT.

50 Douglas Place, Chicago, February 12, 1877.

Editors of the Chicago Medical Journal and Examiner:

*Gentlemen*—Allow me to call attention to a disinfectant, which, being free from disagreeable odor, is an elegant substitute for carbolic acid, chlorine, etc., and may properly supersede them in drawing rooms, sleeping rooms, etc., where odor would be objectionable. I refer to a solution of salicylic acid in *eau de cologne*. In "cologne" this acid is very soluble—my druggist dissolves thirty-six grains to f℥j. Its antiseptic virtues are well attested. It may be used by means of a spray atomizer upon carpets, curtains, clothing, etc.; and the physician will find it the pleasantest method of disinfecting his clothing to prevent the spread through him of the pestilential diseases which are affecting our city at the present time. Many persons—ladies especially—while traveling in horse cars and frequenting churches, halls, etc., drop a few drops of a solution of carbolic acid upon their handkerchiefs. It occurred to me that a good substitute would be an ordinary pungent, in which might be dropped some of the salicylic acid solution. It would certainly be a degree more æsthetic, which is not to be despised in public or private.

Dr. James I. Tucker.

---

The Superintendents of the American Institutions for the Improvement of Idiots and Feeble-minded Children having formed an Association for the more rapid advance and spread of their special part of medical science, resolved, not only to unite their efforts, but to seek the assistance of physicians in general practice who can help them to elucidate the cause of idiocy and kindred affections.

Communications from physicians of the cause of idiocy which have come to their knowledge from reliable witnesses or personal observation, should be sent to the Secretary of the Association, I. N. Kerlin, M.D., Superintendent of the Pennsylvania Training School for Feeble-minded Children, Media, Pennsylvania.

